# Comparative evaluation of antioxidant status, stress, and hepatorenal biomarkers in Nile tilapia *(Oreochromis niloticus)* cultured in In-Pond raceway and traditional aquaculture systems

**DOI:** 10.1007/s10695-025-01531-w

**Published:** 2025-07-04

**Authors:** Salah M. Aly, Osama A. Abd Allah, Noha I. ElBanna, Nahla S. Abdel-Naeim, Noha S. Abdelnaeim, Mohamed Fathi

**Affiliations:** 1 Department of Pathology, Faculty of Veterinary Medicine, Suez Canal University, Ismailia, 41522 Egypt; 2 Department of Clinical Pathology, Faculty of Veterinary Medicine, Suez Canal University, Ismailia, 41522 Egypt; 3 Department of Aquaculture Diseases Control, Fish Farming and Technology Institute, Ismailia, 41522 Egypt; 4 Department of Rangeland, Wildlife and Fisheries Management, College Station, Texas A&M University, Texas, USA; 5 National Institute of Oceanography and Fisheries (NIOF), Cairo, Egypt

**Keywords:** Nile tilapia, IPRS, Antioxidant status, Stress indices, Liver and kidney function biomarkers

## Abstract

The in-pond raceway system (IPRS), is an intensive aquaculture technology that has gained attention in recent years. This study evaluated the comparative effects of In-Pond Raceway Systems (IPRS) and traditional aquaculture systems on oxidative status, stress indices, and biomarkers of liver and kidney function, as well as proteinogram in Nile tilapia *(Oreochromis niloticus)* over 2, 4, and 6 months. The results revealed that serum total antioxidative capacity (TAC) level were significantly lower in the IPRS group compared to the traditional system at all time points (*p* < 0.01 at 2 months, *p* < 0.001 at 4 and 6 months), indicating oxidative stress in the IPRS. Stress markers such as cortisol and glucose levels were significantly elevated in the IPRS group at 4 and 6 months (*p* < 0.05 and *p* < 0.001, respectively), while triglycerides and cholesterol levels were consistently higher in the IPRS group across all time points (*p* < 0.001 for triglycerides, *p* < 0.001 at 2 months and *p* < 0.01 at 4 and 6 months for cholesterol). Liver function biomarkers, including alanine aminotransferase (ALT) and aspartate aminotransferase (AST), showed no significant differences between the two systems, except for a transient increase in ALT at 4 months in the IPRS group (*p* < 0.05). These findings underscore the need for optimized IPRS management strategies, particularly adjusted stocking densities, to mitigate physiological stress and ensure sustainable aquaculture. The traditional system demonstrated lower stress impacts, promoting better health and metabolic stability, offering valuable insights for balanced aquaculture practices.

## Introduction

Aquaculture is among the fastest-growing food production sectors globally, driven by the escalating demand for animal protein and the challenges of ensuring global food security (FAO 2024). Traditional aquaculture systems, while effective in certain contexts, are insufficient to meet the increasing demand for high-quality fish protein. Consequently, the adoption of intensive aquaculture systems has become essential. These systems leverage modern advancements in pond design, feed formulation, and management techniques to enhance production efficiency, yielding higher outputs while optimizing resource use (Komal et al. 2024). Production intensification has thus emerged as a pivotal strategy to ensure a sustainable and consistent supply of aquaculture products (Crab et al. 2012; Subasinghe et al. 2009).

Nile tilapia (*Oreochromis niloticus*) farming represents a cornerstone of the aquaculture industry, characterized by its rapid expansion and profitability. As one of the most widely cultivated fish species, tilapia ranks second only to carp in global production (FAO 2024). Its appeal lies in its exceptional growth rate, high protein content, and ability to thrive under a variety of farming conditions, including intensive systems (Aich et al. 2022). Moreover, tilapia's adaptability to dense stocking and its reproductive efficiency have solidified its position as a preferred species for high-density aquaculture operations (Mtaki et al. 2024). As aquaculture practices evolve, tilapia is anticipated to play an even more significant role in addressing global food demands.

The In-Pond Raceway System (IPRS) has garnered attention as an innovative high-density culture technique designed to maximize productivity while utilizing only a fraction (3%–5%) of the pond area for fish cultivation. This system consists of a rectangular raceway or cage placed within the pond, supported by high aeration to ensure constant water circulation. By confining fish to a smaller, more controllable environment, the IPRS allows for enhanced management of feeding, treatments, and inventory (Roy et al. 2019). However, the system’s high stocking densities present unique challenges, particularly regarding the heightened risk of disease outbreaks and the difficulty of responding to these events within the constrained space.

Environmental stressors such as overcrowding, reduced water quality, and handling practices exacerbate the susceptibility of aquaculture systems to pathogenic threats, including bacteria, viruses, and parasites (Naylor et al. 2021; Lulijwa et al. 2019). These stressors, compounded by intensive farming conditions, undermine the immune system of cultured fish, increasing their vulnerability to infections (Ni et al. 2021; Adineh et al. 2019; Liang et al. 2018; Rosa et al. 2013; Castro et al. 2011). Disease outbreaks, consequently, have become a significant barrier to aquaculture sustainability, leading to substantial economic losses. It is estimated that infectious diseases account for at least 40% of global costs in some aquaculture sectors (FAO 2020; Carpenter 2019; Shinn et al. 2018).

Stocking density is a critical factor influencing the physiology, growth, and welfare of Nile tilapia (*Oreochromis niloticus*) in aquaculture systems. Higher stocking densities have been shown to negatively affect growth performance, feed efficiency, and survival rates, while inducing physiological stress marked by elevated cortisol and glucose levels (Li et al. 2012; Metwaly et al. 2025). Increased stocking density disrupts biochemical markers, including serum protein and ion concentrations, and alters immune responses, as evidenced by changes in inflammatory markers such as TNF-alpha (Metwaly et al. 2025; Sundararajan et al. 2024). Additionally, stocking density impacts digestive enzyme activity and antioxidant responses, with higher densities leading to reduced enzymatic activity and increased oxidative stress (Ni et al. 2021).

This study evaluates the physiological responses of Nile tilapia cultured in IPRS compared to traditional systems, focusing on antioxidant status, stress indices, liver and kidney function biomarkers, and serum proteinogram. As Nile tilapia is one of the most widely cultivated fish species globally, and IPRS represents an emerging intensive farming technique, understanding the health implications of this system is critical for optimizing production and ensuring fish welfare. While previous research has examined individual biomarkers under intensive conditions, this study is among the first to integrate a comprehensive panel of antioxidant, organ function, and stress-related parameters specifically within the IPRS context. By directly comparing these profiles between IPRS and conventional systems, we provide a holistic assessment of fish health under high-density raceway culture. This approach not only elucidates the physiological impacts of intensive farming but also offers novel insights into optimizing such systems to maintain fish health, support disease prevention, and enhance the sustainability of modern aquaculture practices.

## Materials and methods

### Aquaculture systems

#### In-Pond Raceway System (IPRS)

**Fig. 1:**
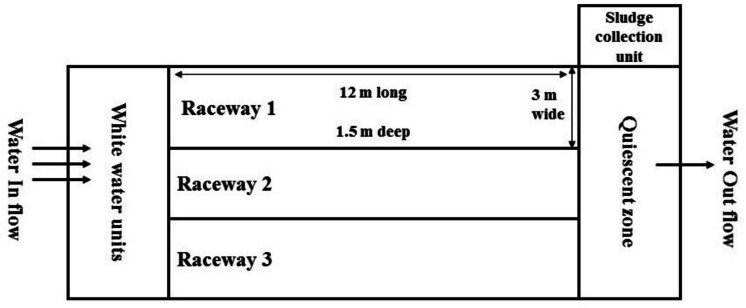
Diagram for the IPRS production system used in this study

The In-Pond Raceway System (IPRS) was created at WorldFish in Abbassa, Abou-Hammad Sharkia, Egypt, for the purpose of tilapia farming. This advanced design features three raceway ponds, each measuring 12 m by 3 m by 1.5 m (Fig. 1), embedded within a larger pond that spans one feddan (4200 m^2^). The raceway ponds function as the main production units for the fish, while the surrounding open water area acts as a zone for treating gas waste. Each pond is equipped with a 1-hp airlift or white-water unit that maintains a constant water flow at speeds of 7–10 cm per second, ensuring effective aeration during the production cycle. The earthen pond is segmented by dikes, which facilitate complete circulation by allowing water to flow through the raceways and around the entire pond before re-entering the system. The stocking density is set at 200 fish per cubic meter in the production areas, thereby optimizing both space and production efficiency. Prior to the commencement of the study, regular monitoring of the IPRS site confirmed that there were no active disease outbreaks.

#### Traditional pond system

In this study, three traditional ponds at WorldFish in Abbassa, Abou-Hammad Sharkia, Egypt, were used as control units. Each pond encompasses an area of 4200 m^2^ (200 × 21 m) and operates under standard pond aquaculture practices, maintaining a stocking density of 4 fish per cubic meter—a norm for such systems in Egypt. Prior to the commencement of the study, both systems were evaluated for any pre-existing conditions, with no significant disease outbreaks observed at that time.

### Feeding management

Fish in both systems were fed a floating commercial feed (SKRETTING®, Egypt) tailored to their growth stage and biomass. During the early growth phase, when the average fish weight was below 100 g, the feed contained 32% crude protein, administered at 5% of the total biomass. Once the fish exceeded 100 g in weight, the protein content in the feed was reduced to 30%, and the feeding rate was adjusted to 3% of the total biomass. Feeding was conducted three times daily at 8:30 am, 11:30 am, and 3:30 pm to ensure consistent nutritional support and promote optimal growth.

### Ethical Approval

The experimental protocol, including fish handling and aquaculture management practices, was reviewed and approved by the Research Ethical Committee of the Faculty of Veterinary Medicine, Suez Canal University (Approval No. 2022001). The study adhered to ethical standards for the care and use of aquatic animals, ensuring that all procedures complied with institutional and international guidelines.

### Water quality assessment

Water quality was assessed throughout the experiment by collecting samples from both IPRS and traditional system ponds. Samples were obtained by completely submerging sterile, dark glass bottles (field-rinsed with pond water) 0.15–0.3 m below the surface, at least one meter from the pond edge, bottles were sealed tightly, maintained on ice, and transported promptly to the laboratory (Boyd 1984). Analysis comprised physicochemical parameters (temperature, pH, dissolved oxygen (DO), ammonia, nitrite, nitrate) measured in situ or immediately upon arrival using a calibrated multiparameter meter and a multiparameter photometer, respectively (Zhang et al. 2022). Microbial analysis, targeting heterotrophic plate counts, was performed using the standardized pour plate method (Clesceri et al. 1998).

### Blood sampling

A total of 90 Nile tilapia *(Oreochromis niloticus)* were sampled throughout the study. The fish were sourced from both IPRS ponds, specifically Raceways 1, 2, and 3, as well as traditional earthen ponds labeled C1, C2, and C3. Sampling was conducted at three time points: 2, 4, and 6 months. During each sampling event, five fish were randomly selected from each pond. The average size of the fish sampled at 2 months was 74.82 ± 15.78 g, at 4 months was 121.94 ± 23.14 g, and at 6 months was 178.31 ± 33.89 g. To minimize stress, the fish were anesthetized with benzocaine at a concentration of 100 mg/L, following the method described by Dawson (1979).

Blood samples were drawn from the caudal peduncle vein using sterile syringes then transferred into plain tubes. The collected blood was allowed to clot at 4 °C and subsequently centrifuged at 1000 × g for 10 min to separate serum. This serum was carefully collected and stored at − 20 °C for the biochemical analysis.

### Serum biochemical parameters

#### Antioxidant status and stress indices

Total antioxidant capacity (TAC) was measured using a commercial kit from LDN (Germany) in accordance with the procedures of Cao et al. (1993). Using the CUSABIO kit (USA), cortisol level was determined as described by Velasco-Santamaría and Cruz-Casallas (2007). The method used to measure serum glucose was outlined by Weissman and Klein (1958). Following the guidelines of Tietz and Berger (1976) and Bucolo and David (1973), respectively, the levels of triglycerides (TG), and total cholesterol (TC) were measured. Glucose, triglycerides and total cholesterol were tested using Spectrum kits (Egypt).

#### Liver function biomarkers and proteinogram

Serum alanine aminotransferase (ALT) and aspartate aminotransferase (AST) activities were determined using the method described by Reitman and Frankel (1957) to evaluate the liver functions. Serum total proteins and albumin concentrations were assessed in accordance with Doumas et al. (1971) and Henry (1964), respectively. Commercially accessible kits from Spectrum (Egypt) were used to measure all the parameters. The calculation of the globulin level and albumin/globulin (A/G) ratio was conducted according to Duran et al. (2014) and Busher (1990), respectively.

#### Kidney function biomarkers

Serum urea and creatinine were tested as described by Tietz and Andresen (1986) and Tietz (1995), respectively using Spectrum commercial kits (Egypt).

### Statistical analysis

The collected data were statistically analyzed using the Statistical Package for the Social Sciences (SPSS) software, version 27.0 (SPSS Inc., USA). All data were tested for normality using the Shapiro–Wilk test and for homogeneity of variances (homoscedasticity) using Levene's test. The data met the assumptions for parametric analyses (normality and homoscedasticity, *p* > 0.05). Therefore, parametric tests were employed: Descriptive statistics were expressed as mean values ± standard error of the mean (S.E.M.) to summarize the data. To identify significant differences among groups, independent T- test, one-way, and two-way analysis of variance (ANOVA) tests were employed, depending on the experimental design and the factors being analyzed. Following the ANOVA, Duncan’s Multiple Range Test (Duncan 1955) was performed as a post hoc analysis to determine specific group differences. Statistical significance was defined as a p-value of less than 0.05.

## Results

### Water quality assessment

Water quality parameters in traditional and IPRS ponds are summarized in Table 1. Dissolved oxygen (DO) was significantly higher in IPRS ponds (7.15 ± 0.03 mg/L) compared to traditional ponds (4.22 ± 0.09 mg/L) (*P* < 0.001). No statistically significant differences (*P* > 0.05) were observed for pH, temperature, total ammonia nitrogen (TAN), unionized ammonia, nitrate, nitrite, or total plate count (TPC) between the two pond systems, although numerical trends were evident for some parameters (e.g., higher mean nitrate in IPRS, lower mean TPC in IPRS).

**Table 1:** Water quality indicators in traditional and IPRS ponds

Parameters	Traditional pond	IPRS ponds	Sig (2-tailed)	95% Confidence Interval of the Difference	
				Lower	Upper
pH	8.052 ± 0.30	7.901 ± 0.17	0.64	−0.83	0.53
DO (mg/l)	4.22 ± 0.09^**b**^	7.15 ± 0.03^**a**^	< 0.001	2.77	3.09
Temperature ˚C	25.62 ± 2.03	25.62 ± 1.20	1	−4.78	4.79
TAN (mg/l)	0.30 ± 0.10	0.26 ± 0.06	0.705	−0.27	0.19
Unionized ammonia(mg/l)	0.03 ± 0.02	0.02 ± 0.01	0.227	−0.05	0.01
Nitrate (mg/l)	16.55 ± 8.84	24.08 ± 10.09	0.644	−27.66	42.70
Nitrite (mg/l)	0.005 ± 0.002	0.087 ± 0.054	0.176	−0.047	0.21
TPC (CFU/ml)	9.78 ± 7.02	3.32 ± 1.29	0.414	−25.77	12.86

D.O dissolved oxygen, TAN total ammonia nitrogen, TPC total plate count. Data are expressed as mean ± SE. Different superscripts (a-b) in the same row indicate highly significant difference (*P* < 0.001)

### Serum biochemical parameters

#### Antioxidant status and stress indices

**Fig. 2:**
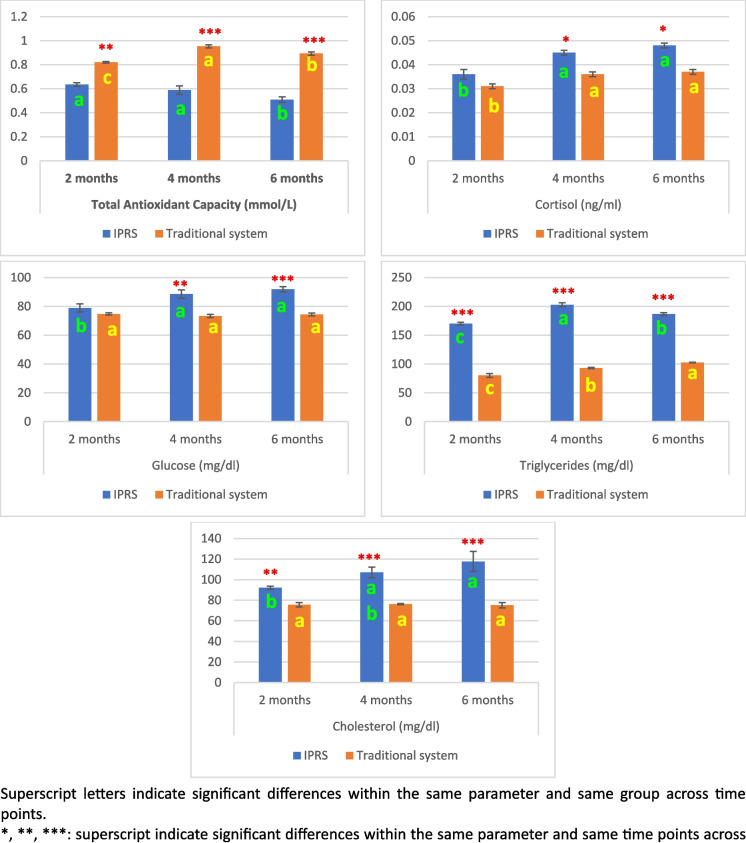
Serum Total Antioxidant Capacity (TAC), and stress indices of Nile tilapia fish reared at IPRS and traditional culture system at 2, 4, and 6 months

As shown in Fig. 2, Total Antioxidant Capacity (TAC) levels were significantly higher in the traditional pond compared to the IPRS at all measured time points, with significance *p* < *0.01* at 2 months and *p* < *0.001* at 4, and 6 months. Within the IPRS, TAC levels showed a significant decrease at 6 months compared to both at 2 and 4 months, with no significant difference between 2 and 4 months. In the traditional group had the highest level at 4 months, followed by 6 months and then 2 months.

Serum cortisol levels in the IPRS were significantly higher (*p* < 0. 05) at 4, and 6 months compared to the traditional system. Furthermore, glucose levels in the IPRS showed significant increase (*p* < *0. 05*) at 4 months and highly significant increase (*p* < *0. 001*) at 6 months compared to the traditional system. At 2 months, cortisol and glucose levels didn’t show significant changes between the two groups. Cortisol and glucose levels within the IPRS increased dramatically at 4 and 6 months compared to 2 months. Conversely, in the traditional pond, the cortisol level rose gradually over time, reaching a notable peak at 4 and 6 months as compared to 2 months, while glucose level didn’t exhibit significant alterations at any of the time points.

Triglycerides levels (TG) were consistently and highly significant increase (*p* < *0.001*) in the IPRS in comparison to the traditional system across all time points. Within the IPRS, TG levels peaked at 4 months followed by 6 and then 2 months. In contrast, the traditional system showed its highest TG levels at 6 months followed by 4 and then 2 months.

The total cholesterol levels in the IPRS we highly significantly increased (*p* < *0.001*) at 2 months and significant increase (*p* < *0. 01*) at 4, and 6 months compared the traditional system. Within the IPRS, the cholesterol levels markedly decreased at 2 months as compared to 6 months, while there was no significant difference between 4 and 6 groups. In contrast, there was no notable change in the traditional system at all the time points.

Two-way ANOVA indicated that the rearing system was the predominant factor influencing the antioxidant status and the stress indices. Highly significant variance (*P* < *0.001*) attributable to the system were observed for TAC, cortisol, glucose, TG, and TC levels. The effect of time and the system- time interaction had comparatively smaller contributions, although statistically significant effects were noted for some parameters. Specifically, time had a highly significant effect (*P* < *0.001*) on cortisol and TG levels, but not on TAC, glucose, and cholesterol levels. The interaction between system and time significantly influenced TAC (*P* < *0.01*) and TG (*P* < *0.001*) levels, with no significant interaction effects observed in cortisol, glucose, and cholesterol levels (Table 2).

**Table 2:** Two-way ANOVA: The effect of system, time and their interaction on serum TAC, and stress indices of Nile tilapia fish

Item	System	Time	System * Time
TAC	***	NS	**
Cortisol	***	***	NS
Glucose	***	NS	NS
Triglycerides	***	***	***
Cholesterol	***	NS	NS

The significance level was determined as follows: NS (nonsignificant) indicates no statistically significant effect, *represents a significance level of *p* ≤ 0.05, **represents *p* ≤ 0.01, and ***represents *p* ≤ 0.001

#### Liver function biomarkers and proteinogram

**Fig. 3:**
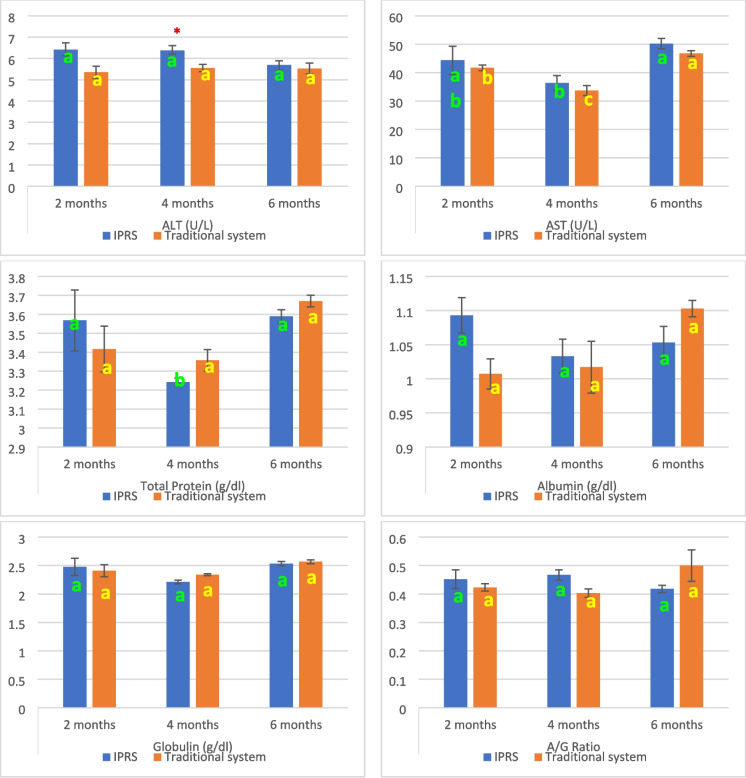

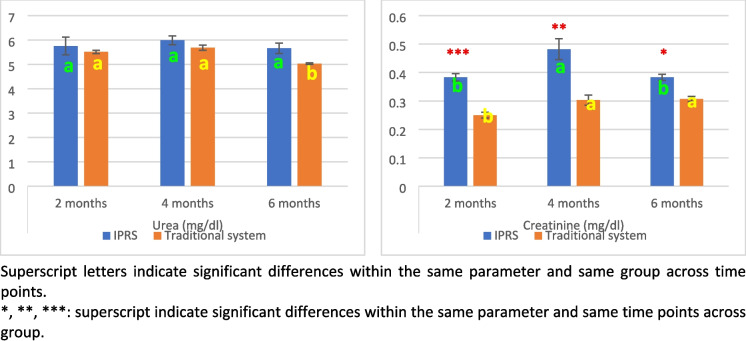
Serum liver enzymes, proteinogram and kidney function biomarkers of Nile tilapia fish reared at IPRS and traditional culture system at 2, 4, and 6 months

Alanine aminotransferase (ALT) activity was comparable between the IPRS and traditional systems at 2 and 6 months (Fig. 3). However, at 4 months, ALT activity was significantly higher (*p* < *0. 05*) in the IPRS. Furthermore, no significant change in ALT activity were observed over time within either rearing system.

Aspartate aminotransferase (AST) activity did not differ significantly between IPRS and traditional system at any time points. Within the IPRS, AST activity highest at 6 months, followed by 4 months, and was not significantly different at 2 months from 4 and 6 months. In the traditional pond, AST activity revealed a notable rise at 6 months, followed by 2 months, and then 4 months. T. proteins showed no significant variations between IPRS and traditional pond at all time points. In the IPRS, T. proteins increased significantly at 2 and 6 months as compared to 4 months. Conversely, the T. proteins levels in the traditional pond didn’t exhibit significant changes at all time points. Albumin, globulin and A/G ratio didn’t exhibit significant variations between IPRS and traditional groups at all time points. Within each group, these parameters didn’t show significant changes at all time points.

As shown in Table (3), there was significant variance in ALT activity (*P* < *0.01*) under the effect of the system, while no statistical variances were detected in AST, T. proteins, albumin, globulin, and A/G ratio. On the other hand, under the effect of time, there was significant variance in AST activity, total proteins (*P* < *0.01*), and globulin, (*P* < *0.05*) levels, while no statistical variances were detected in ALT, albumin, and A/G ratio. However, the interaction between system and time didn’t result in significant variances in ALT and AST activities, total proteins, albumin, and globulin levels as well as A/G ratio.

#### Kidney function biomarkers

Urea levels did not differ significantly between the IPRS and traditional systems at any time point (Fig. 3). Within the IPRS, urea levels remained consistent with no significant changes over time. However, in the traditional system, levels showed a significant decrease over time, with higher levels observed at 2 and 4 months compared to 6 months. Serum creatinine levels were significantly higher in the IPRS compared to the traditional pond at all time points, with a highly significant increase at 2 months (*p* < 0.001), a significant increase at 4 months (*p* < 0.01), and a significant increase at 6 months (*p* < 0.05). Within the IPRS, creatinine levels peaked at 4 months and were significantly lower at 2 and 6 months. In contrast, in the traditional pond, creatinine levels increased significantly over time, with higher levels at 4 and 6 months compared to 2 months.

The two-way ANOVA (Table 3) revealed a highly significant effect of the rearing system on creatinine levels (*P* < 0.001), but not on urea levels. Time significantly affected creatinine levels (*P* < 0.05) but had no significant effect on urea. There were no significant interaction effects between system and time for either urea or creatinine levels.

**Table 3:** Two-way ANOVA: The effect of system, time and their interaction on serum liver enzymes, proteinogram and kidney function biomarkers of Nile tilapia fish

Item	System	Time	System * Time
ALT	**	NS	NS
AST	NS	**	NS
Total Proteins	NS	**	NS
Albumin	NS	NS	NS
Globulin	NS	*	NS
A/G Ratio	NS	NS	NS
Urea	NS	NS	NS
Creatinine	***	*	NS

The significance level was determined as follows: NS (nonsignificant) indicates no statistically significant effect, *represents a significance level of *p* ≤ 0.05, **represents *p* ≤ 0.01, and ***represents *p* ≤ 0.001

## Discussion

This study provides a comparative evaluation of the physiological responses of Nile tilapia (*Oreochromis niloticus*) cultured in the In-Pond Raceway System (IPRS) versus the traditional aquaculture system focusing on antioxidant status, stress indices, liver and kidney function biomarkers, and proteinogram over 2, 4, and 6 months. The results reveal significant differences between the two systems, in several physiological parameters, particularly related to stress and antioxidant capacity, which are likely influenced by the high stocking density and environmental conditions inherent to the IPRS.

Antioxidant biomarkers are used to identify the effects of different environmental stresses on aquatic organisms (Hook et al. 2014). In our study, the significantly lower total antioxidant capacity (TAC) in the IPRS compared to the traditional pond system at all time points (*p* < 0.01 at 2 months, *p* < 0.001 at 4 and 6 months) indicates a compromised antioxidant defense system under high-density conditions. This aligns with findings by Metwaly et al. (2025), who reported that Nile tilapia fingerlings exposed to high salinity and ammonia levels exhibited reduced antioxidant enzyme activities, suggesting oxidative stress due to environmental stressors. Similarly Zaki et al. (2023), observed lower TAC level in *Pangasianodon hypophthalmus* under high stocking density, attributing it to chronic stress of overcrowding, which generates reactive oxygen species (ROS) and overwhelms antioxidant defenses. The progressive trend in our study, the TAC reduction was most pronounced at 4 and 6 months in the IPRS, suggesting a time-dependent escalation of oxidative stress, possibly due to prolonged exposure to high stocking density. The lack of significant TAC differences at 2 months may reflect fish were still small in size and not yet affected by overcrowding, and an initial adaptation period, with oxidative stress becoming more pronounced over time as metabolic demands increase.

Cortisol and glucose levels, are two of the most prevalent markers of fish stress (Wang et al. 2020). According to our results, cortisol and glucose levels were significantly higher in the IPRS at 4 and 6 months (*p* < 0.05 and *p* < 0.001, respectively), compared to the traditional system, with no significant differences at 2 months. These findings are consistent with Metwaly et al. (2025), who reported elevated cortisol and glucose levels in Nile tilapia under high stocking densities, indicating a stress response triggered by crowding. The time-specific increase in these stress markers suggests that the physiological burden of high-density conditions in the IPRS accumulates over time, potentially due to sustained competition for resources and space. In contrast, the traditional pond system, with a lower stocking density of 4 fish/m^3^, maintained stable cortisol and glucose levels, suggesting a less stressful environment. Luz et al. (2025) also noted higher cortisol levels in proactive Nile tilapia under high-density conditions, supporting the notion that stress in IPRS may be linked to social and spatial constraints.

Triglycerides (TG) and total cholesterol (TC) levels are commonly used indicators for evaluating fish stress and are associated with lipid metabolism and energy (Jia et al. 2022). According to our findings, TG and TC levels were significant elevated in the IPRS at all the time points. (*p* < 0.001 at 2 months, *p* < 0.01 at 4 and 6 months), indicating altered lipid metabolism likely driven by stress-induced energy mobilization. These results corroborate those of Khan et al. (2023) reported elevated glucose and TG levels in Nile tilapia under high-density stress, linking these changes to increased metabolic demands. The absence of significant TG and TC differences at earlier time points in the traditional system suggests that lower stocking densities mitigate these metabolic disruptions, promoting lipid homeostasis.

The lack of significant differences in cortisol and glucose at 2 months between systems may be attributed to the initial acclimatization phase, where fish in the IPRS had not yet experienced prolonged stress. Additionally, water quality parameters, such as dissolved oxygen (DO) and ammonia levels, which were not monitored in this study, could contribute to these differences. For instance, Qiang et al. (2012) demonstrated that suboptimal water quality (e.g., low DO or high ammonia) exacerbates stress responses in Nile tilapia, potentially amplifying the observed effects in the IPRS due to its higher stocking density and confined water circulation.

Alanine aminotransferase (ALT) and Aspartate aminotransferase (AST) enzymes are mostly found in the liver and are crucial markers of hepatic function (Liu et al. 2015). According to our results, no significant differences were observed in alanine aminotransferase (ALT) activity between the IPRS and traditional systems at 2 and 6 months, but a significant increase (*p* < 0.05) was noted in the IPRS at 4 months. Aspartate aminotransferase (AST) activity showed no significant differences between systems at any time point, suggesting that liver function may remain relatively stable under moderate stress. However, the transient increase in ALT at 4 months in the IPRS could indicate temporary hepatic stress, possibly due to peak metabolic activity or water quality fluctuations during this period. Metwaly et al. (2025) reported elevated ALT and AST in Nile tilapia under high ammonia and salinity stress, suggesting that environmental factors in the IPRS, such as ammonia accumulation, may contribute to this temporary elevation. Our results agree with those of Wang et al. (2019), who reported that fish raised at low and high stocking densities did not significantly differ in ALT and AST activities.

Total proteins, albumin, globulin, and the albumin/globulin (A/G) ratio showed no significant differences between the two systems at any time point, indicating that protein metabolism was not significantly impacted by the culture system. These results are consistent with Arafa et al. (2024) reported stable protein profiles in Tilapia species under varying stocking densities, suggesting that tilapia species may maintain protein homeostasis under moderate stress conditions. The lack of significant differences in proteinogram parameters suggests that both systems support adequate protein synthesis, although the IPRS may impose greater metabolic demands over time.

Serum urea levels showed no significant difference between the IPRS and traditional systems at any of the time points, suggesting that kidney function was not significantly impaired by the high stocking density in the IPRS. However, creatinine levels were significantly higher in the IPRS, through within normal ranges of the Nile tilapia (El-Barbary 2017; Sayed et al. 2024). These findings align with Shaban et al. (2022) who reported no significant changs in urea, and creatinine levels of Nile tilapia across different stocking densities. indicating robust kidney function resilience. The elevated creatinine in the IPRS may reflect increased muscle catabolism due to stress, as suggested by Ni et al. (2021), but the levels remaining within normal ranges suggest minimal impact on renal health.

### Role of water quality and system-specific factors

The observed physiological differences between the IPRS and traditional systems may be partly attributed to intrinsic differences in water quality and system design. The IPRS, with a stocking density of 200 fish/m^3^, likely experiences higher ammonia and lower DO levels compared to the traditional system (4 fish/m^3^), as noted in studies like Suresh and Lin (1992), which reported lower ammonia and nitrite levels at optimal stocking densities in green-water systems. Poor water quality in high-density systems can exacerbate oxidative stress and metabolic disruptions, as evidenced by the reduced TAC and elevated stress markers in the IPRS. The confined raceway design of the IPRS, despite high aeration, may limit water exchange compared to the open traditional ponds, potentially leading to accumulation of metabolic waste. Qiang et al. (2012) highlighted the synergistic effects of high stocking density and poor water quality on Nile tilapia stress responses, supporting the hypothesis that water quality differences contribute to the observed physiological impacts.

The time-specific responses observed in this study, such as the delayed onset of significant cortisol and glucose differences at 4 and 6 months, may reflect the cumulative effects of chronic stress and environmental factors in the IPRS. The traditional system’s lower stocking density and larger water volume likely provide a more stable environment, reducing stress and supporting metabolic homeostasis. Future studies should include detailed water quality monitoring (e.g., DO, ammonia, pH) to quantify these effects and elucidate their contributions to physiological outcomes.

### Implications and practical considerations

The dominance of treatment effects observed in the two-way ANOVA analysis highlights the profound influence of culture systems on physiological parameters in Nile tilapia. IPRS, while advantageous for high production, imposes chronic stress, evident from elevated cortisol, glucose, and triglyceride levels and reduced antioxidant capacity. These physiological disruptions may have long-term implications for fish health, growth, and immune competence. On the other hand, the traditional system offers a lower-stress environment, promoting better metabolic stability and immune function.

To optimize IPRS performance, strategies to, mitigating stressors are essential. Improved aeration, regular water exchange, and optimized stocking densities (e.g., reducing from 200 fish/m^3^ to levels closer to 50–113 fish/m^3^, as suggested by Ni et al. 2021) could alleviate oxidative stress and metabolic disruptions. The use of dietary supplements, such as elderberry extract (Khan et al. 2023) or bitter orange peel powder (Gressler et al. 2024), has shown promise in modulating stress and enhancing antioxidant capacity in Nile tilapia, offering potential solutions for IPRS management. Additionally, biofloc technology, as explored by Khanjani et al. (2024), could improve water quality and reduce stress in high-density systems by enhancing microbial nutrient cycling.

The lack of significant differences in some parameters, such as urea and proteinogram, suggests that Nile tilapia is resilient to certain stressors, but the time-specific responses highlight the need for longitudinal studies to assess chronic effects. Further research is needed to investigate the long-term impacts of IPRS on growth performance, disease resistance, and reproductive outcomes, as well as to establish reference ranges for physiological parameters specific to IPRS-cultured Nile tilapia. Such studies will provide critical insights for developing sustainable aquaculture practices that balance productivity with fish welfare.

## Conclusion

This study demonstrates that the In-Pond Raceway System (IPRS) imposes significant physiological stress on Nile tilapia (*Oreochromis niloticus*) compared to the traditional pond system, primarily due to high stocking density and associated environmental factors. The reduced total antioxidant capacity (TAC) and elevated cortisol, glucose, and triglyceride levels in the IPRS at 4 and 6 months indicate a time-dependent escalation of oxidative stress and metabolic disruptions, which were less pronounced at 2 months when fish were smaller and less affected by overcrowding. These findings suggest that while Nile tilapia exhibit initial resilience during an acclimatization phase, prolonged exposure to high-density conditions in the IPRS compromises antioxidant defenses and increases stress, potentially affecting long-term health, growth, and immune function. In contrast, the traditional pond system, with its lower stocking density, supports greater metabolic stability and reduced stress, highlighting its suitability for maintaining fish welfare.

The observed differences in physiological parameters, such as transient elevations in alanine aminotransferase (ALT) and consistently higher creatinine levels in the IPRS, underscore the influence of system-specific factors like water quality and spatial constraints. Although kidney function (urea) and protein metabolism (total proteins, albumin, globulin) remained relatively stable across both systems, the cumulative stress in the IPRS suggests the need for management strategies to mitigate these effects. Optimizing stocking densities, enhancing aeration, implementing regular water exchange, and incorporating dietary supplements or biofloc technology could alleviate stress and improve antioxidant capacity in IPRS, thereby enhancing fish health and productivity.

These results emphasize the trade-offs between high-yield aquaculture systems like IPRS and the physiological well-being of Nile tilapia. Future research should focus on longitudinal studies to assess the chronic impacts of IPRS on growth, disease resistance, and reproduction, while incorporating detailed water quality monitoring to better understand environmental contributions to stress. Establishing system-specific reference ranges for physiological parameters will further aid in developing sustainable aquaculture practices that balance productivity with fish welfare, ensuring the long-term viability of intensive systems like IPRS.

## Data Availability

The datasets used and/or analyzed during the current study are available from the corresponding author upon reasonable request.
